# Molecular Identification of Rickettsial Endosymbionts in the Non-Phagotrophic Volvocalean Green Algae

**DOI:** 10.1371/journal.pone.0031749

**Published:** 2012-02-21

**Authors:** Kaoru Kawafune, Yuichi Hongoh, Takashi Hamaji, Hisayoshi Nozaki

**Affiliations:** 1 Department of Biological Sciences, Graduate School of Science, University of Tokyo, Hongo, Bunkyo-ku, Tokyo, Japan; 2 Department of Biological Sciences, School of Bioscience and Biotechnology, Tokyo Institute of Technology, Ookayama, Meguro-ku, Tokyo, Japan; 3 Department of Botany, Graduate School of Science, Kyoto University, Oiwake-cho, Kita-shirakawa, Sakyo-ku, Kyoto, Japan; University of Poitiers, France

## Abstract

**Background:**

The order Rickettsiales comprises Gram-negative obligate intracellular bacteria (also called rickettsias) that are mainly associated with arthropod hosts. This group is medically important because it contains human-pathogenic species that cause dangerous diseases. Until now, there has been no report of non-phagotrophic photosynthetic eukaryotes, such as green plants, harboring rickettsias.

**Methodology/Principal Findings:**

We examined the bacterial endosymbionts of two freshwater volvocalean green algae: unicellular *Carteria cerasiformis* and colonial *Pleodorina japonica*. Epifluorescence microscopy using 4′-6-deamidino-2-phenylindole staining revealed the presence of endosymbionts in all *C. cerasiformis* NIES-425 cells, and demonstrated a positive correlation between host cell size and the number of endosymbionts. Strains both containing and lacking endosymbionts of *C. cerasiformis* (NIES-425 and NIES-424) showed a >10-fold increase in cell number and typical sigmoid growth curves over 192 h. A phylogenetic analysis of 16 S ribosomal (*r*)RNA gene sequences from the endosymbionts of *C. cerasiformis* and *P. japonica* demonstrated that they formed a robust clade (hydra group) with endosymbionts of various non-arthropod hosts within the family Rickettsiaceae. There were significantly fewer differences in the 16 S *r*RNA sequences of the rickettsiacean endosymbionts between *C. cerasiformis* and *P. japonica* than in the chloroplast 16 S *r*RNA or 18 S *r*RNA of the host volvocalean cells. Fluorescence *in situ* hybridization demonstrated the existence of the rickettsiacean endosymbionts in the cytoplasm of two volvocalean species.

**Conclusions/Significance:**

The rickettsiacean endosymbionts are likely not harmful to their volvocalean hosts and may have been recently transmitted from other non-arthropod organisms. Because rickettsias are the closest relatives of mitochondria, incipient stages of mitochondrial endosymbiosis may be deduced using both strains with and without *C. cerasiformis* endosymbionts.

## Introduction

The order Rickettsiales (class Alphaproteobacteria) comprises Gram-negative obligate intracellular bacteria (rickettsias) that are unable to reproduce or survive in the long term outside their host eukaryotic cells. Among them, the family Rickettsiaceae is medically important because it contains human-pathogenic species that cause dangerous diseases [1]. This family is currently composed of two genera, *Rickettsia* and *Orientia*; in both, bacteria are mainly associated with arthropod hosts and often infect vertebrates [1]. Infection with *Rickettsia* and *Orientia* in vertebrates is mediated by blood-sucking arthropods such as ticks and lice [2]. Due to their great medical significance, the molecular mechanisms underlying rickettsial infections have been investigated extensively [3], [4]. In addition, because they are the closest relatives of the ancestral bacterium of mitochondria, rickettsias have also been the focus of many studies on eukaryotic evolution [5].

Recently, several Rickettsiaceae species associated with non-arthropod hosts have been reported in the cells of various organisms, such as leeches [6], [7], hydras [8], amoebas [9], haplosporidians [10], and ciliates [11]–[13]. These rickettsias are phylogenetically placed in separate positions within the Rickettsiaceae [13], [14]. Moreover, endosymbionts closely related to the Rickettsiaceae have been discovered within the cells of the plastid-lacking heterotrophic euglenid flagellate *Petalomonas sphagnophila* [15]. Little is known about the virulence or contribution of these endosymbionts to their non-arthropod hosts [16]. To date, rickettsial endosymbionts have not been reported within the cells of non-phagotrophic, photosynthetic eukaryotes, such as primary photosynthetic eukaryotes, or Archaeplastida (green plants [land plants and green algae], red algae, and glaucophytes). Although lacking evidence for their presence within the host cells, they have been detected in the phloem of the papaya tree; since they are thought to cause Bunchy Top disease, they may well penetrate cells [17].

The order Volvocales comprises flagellate green algae that are mainly found in freshwater environments [18], including unicellular *Chlamydomonas* and multicellular *Volvox*. Presence of endosymbiotic bacteria within the cytoplasm was first reported in *Volvox carteri* by transmission electron microscopy (TEM) [19]. The endosymbionts were rod-shaped and localized in the cytoplasm of the host cells without encompassing membranous structures, as found in other bacterial endosymbionts [19]. Similar endosymbiotic bacteria were subsequently found in other volvocaleans, including two colonial species, *Pleodorina japonica* and *Eudorina illinoisensis*, and the unicellular *Carteria cerasiformis*, by TEM and/or 4′-6-diamidino-2-phenylindole (DAPI) staining [20]–[22]. However, the molecular identities and phylogenies of these bacterial endosymbionts remain unresolved.

In the present study, we investigated the molecular and biological characteristics of bacterial endosymbionts in the cytoplasm of the Volvocales. We used four related species of the unicellular genus *Carteria*, because previous studies demonstrated the presence of strains both with and without bacterial endosymbionts within a closely related lineage of *Carteria* [22], [23] (Figure S1). Our results suggest that the endosymbionts belong to the family Rickettsiaceae; this is the first report of Rickettsiales endosymbionts harbored within photosynthetic eukaryotic cells.

## Results

### Observations of endosymbionts by DAPI staining

In epifluorescence microscopy and DAPI-staining analyses, DNA showed light blue fluorescence and chloroplasts showed red fluorescence in all *Carteria* strains examined (Table 1 and Figure S1). Based on previous observations of *Carteria* vegetative cells by TEM [22], weak fluorescence in the periphery of the cytoplasm and amorphous fluorescence within red chloroplasts can be assigned to mitochondrial and chloroplast nucleoids, respectively. In addition to these signals, rod-shaped small bodies (1–2 µm long) within the cytoplasm of *C. cerasiformis* NIES-425 emitted strong fluorescence signals (Figure S2). These bodies were present mainly in the periphery of the cytoplasm outside chloroplasts or around the nucleus. They could be distinguished from chloroplasts and mitochondrial nucleoids by their strong fluorescence, rigid rod-shape, and distribution pattern in the cytoplasm. The epifluorescence microscopic features of the endosymbionts in *C. cerasiformis* NIES-425 were essentially the same as those in *Pleodorina japonica* [20]. In contrast, DAPI-stained cells of *C. cerasiformis* NIES-424 and other *Carteria* species contained no rod-shaped endosymbionts in their cytoplasm (Figure S3), as observed by TEM [22].

**Table 1:** List of the volvocalean strains and their presence or absence of rickettsial endosymbionts examined in this study.

Taxon	Strain designation	Rickettsial endosymbionts
*Carteria cerasiformis*	NIES^a^-424	absent^c^^,^ d
	NIES-425	present^c^^,^ d
*Carteria inversa*	NIES-422	absent^c^^,^ d
	NIES-423	absent^c^^,^ d
*Carteria crusifera*	NIES-421	absent^c^^,^ d
	NIES-630 (UTEX^b^ 432)	absent^c^^,^ d
*Carteria eugametos*	NIES-631	absent^c^
	NIES-632	absent^d^
	NIES-633	absent^d^
	NIES-634 (UTEX 2161)	absent^c^^,^ d
	NIES-635	absent^c^^,^ d
	NIES-636 (UTEX 1032)	absent^c^^,^ d
*Pleodorina japonica*	NIES-577	present^c^^,^ e

a Microbial Culture Collection at the National Institute for Environmental Studies [24].

b Culture Collection of Algae at the University of Texas at Austin [50].

c Determined in this study.

d Based on TEM by Nozaki et al. [22].

e Based on TEM and DAPI-staining by Nozaki et al. [20].

**Figure 1:**
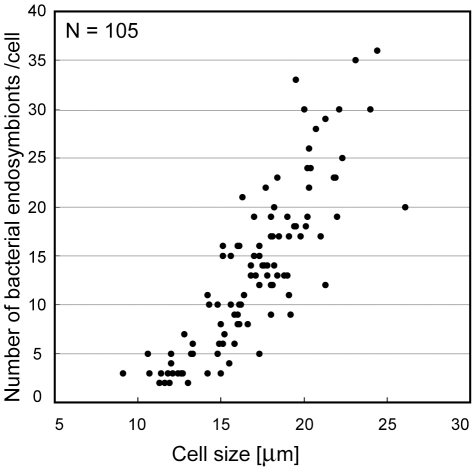
Comparison of host cell size and the number of endosymbionts in *Carteria cerasiformis* NIES-425. Cell size (longitudinal axis) is the diameter of cells fixed and squashed by coverglasses for observation. For details, see Materials and Methods. The graph shows a representative of three determinations; cells were fixed 14 h after the beginning of the light period. *N* = 105, Pearson correlation coefficient (*r*) = 0.84. Two other results are shown as supporting information (Figure S4).

Bacterial endosymbionts were present in all the examined cells of *C. cerasiformis* NIES-425 with various sizes (Figure 1). Based on our measurements, there was a positive correlation (Pearson correlation coefficient = 0.76–0.84) between host cell size and the number of bacterial endosymbionts in all three preparations and at varying timepoints (Figures 1 and S4).

### Growth measurement

Both *C. cerasiformis* NIES-424 and NIES-425 exhibited a more than 10-fold increase in cell number and typical sigmoid growth curves over 192 h after inoculation to new medium (Figure S5). However, growth of *C. cerasiformis* NIES-424 was faster than that of *C. cerasiformis* NIES-425 (Figure S5). A *t*-test of the difference in cell density per culture tube at 192 h showed a significant difference (*P*<0.05) between the two strains.

### Phylogenetic analysis of bacterial endosymbionts based on 16 S *r*RNA

The majority of the endosymbiont 16 S *r*RNA genes of *C. cerasiformis* NIES-425 (1422 bp; AB688628) and *P. japonica* NIES-577 (1399 bp; AB688629) were sequenced (for details, see Materials and Methods). A BLASTn search (http://www.ncbi.nlm.nih.gov/) indicated that the endosymbiont of *C. cerasiformis* NIES-425 is most closely related to the rickettsiacean endosymbiont of the marine ciliate *Diophrys appendiculata* [11] and that of *P. japonica* NIES-577 to the uncultured bacterium clone 214 from Dongping Lake, China (Table S1).

**Figure 2:**
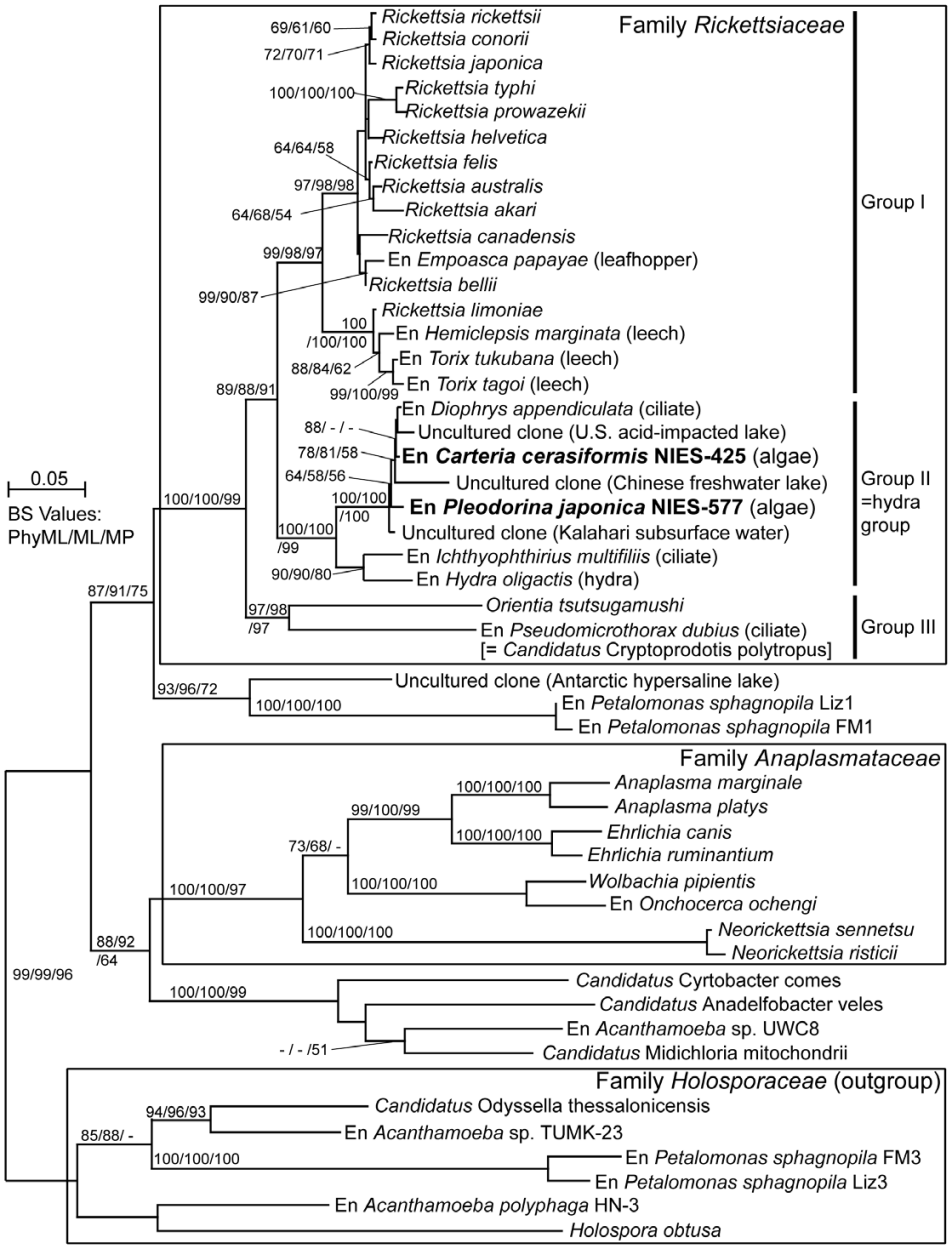
Phylogenetic positions of the endosymbionts of two volvocalean species. The tree was inferred by the PhyML method (TrN+GAMMA+I model) based on 47 16 S *r*RNA gene sequences from bacteria, endosymbionts of (En) eukaryotic hosts, and environmental samples related to the family Rickettsiaceae. The sequences determined in this study are indicated by boldface. Bootstrap values (≥50%) of PhyML, ML (by PAUP*), and MP are indicated to the left, middle, and right of the nodes, respectively. The scale bar shows 0.05 nucleotide substitutions per position. Sequences belonging to the family Holosporaceae were designated as the outgroup. Accession numbers of sequences are shown in supporting information (Table S1). The hydra group refers to Weinert et al. [14].

Phylogenetic analysis of 47 Rickettsiales bacteria based on 16 S *r*RNA gene sequences (Table S1) showed that endosymbionts from the volvocalean species were positioned within the family Rickettsiaceae (Figure 2). The Rickettsiaceae was divided into three robust monophyletic groups (I–III) with ≥97% bootstrap values. Group I was mainly composed of arthropod-associated *Rickettsia* species, including an endosymbiont of the leafhopper *Empoasca papayae* (a possible pathogen of the land plant [17]), and leech-associated *Rickettsia* species [6], [7]. Group II was sister to group I and composed of non-arthropod-associated endosymbionts and environmental sequences, corresponding to the hydra group [14]. Group II was divided into two sister sub-clades: one composed of endosymbionts from the parasitic ciliate *Ichthyophthirius multifiliis* [12] and the metazoan *Hydra oligactis* [8], and the other containing endosymbiont sequences from *C. cerasiformis* NIES-425, *P. japonica* NIES-577, and the marine ciliate *D. appendiculata*, as well as three environmental sequences originating from waters of an acid-impacted lake, a freshwater lake, and the desert (Table S1). Although with only weak or moderate support (58–81% bootstrap values), two endosymbionts from the volvocalean species were non-monophyletic; the endosymbiont of *C. cerasiformis* NIES-425 was more closely related to that of *D. appendiculata* than that of *P. japonica* NIES-577. Group III contained *Orientia tsutsugamushi* and the endosymbiont (*Candidatus* Cryptoprodotis polytropus) of the freshwater ciliate *Pseudomicrothorax dubius* [13], and was positioned most basally within the Rickettsiaceae. Just outside the Rickettsiaceae, a lineage including endosymbionts of the plastid-lacking euglenid *Petalomonas sphagnophila* [15] was positioned. The endosymbionts from the two volvocalean species showed 92.5–93.6% sequence similarity with the bacteria in group I, 95.5–99.5% with other group II bacteria, and 88.7–89.1% with those in group III.

The difference in 16 S *r*RNA sequences of the endosymbionts between the hosts *C. cerasiformis* NIES-425 and *P. japonica* NIES-577 was 0.6%, whereas those in chloroplast 16 S *r*RNA and nuclear 18 S *r*RNA were 7–12% (Table 2).

**Table 2:** Comparison of nucleotide differences in three small *r*RNA subunits (SSU *r*RNA).

	GenBank/EMBL/DDBJ accessionnumbers		Nucleotide differences		
SSU *r*RNA	*Carteria cerasiformis* NIES-425	*Pleodorina japonica* NIES-577	Divergence	Counts (gaps)	Total nucleotides aligned
Bacterial 16 S	AB688628^a^	AB688629^a^	0.57%	8 (0)	1399
Chloroplast 16 S	AB688625^a^	AB688626^a^	12%	140 (42)	1174
18 S	AB688624^a^	AB688627^a^	7.2%	128 (43)	1775

a Sequenced in this study using primers listed in Table S2 [31], [46], [47], [51], [52].

### Fluorescence *in situ* hybridization (FISH)

**Figure 3:**
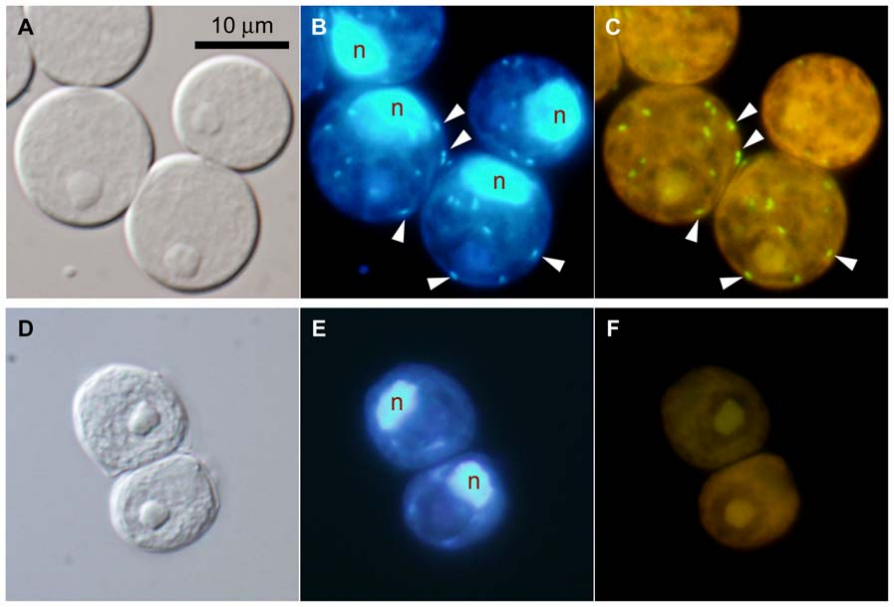
FISH identification of rickettsiacean endosymbionts in *Carteria cerasiformis* cells. **A–C.** *C. cerasiformis* NIES-425. **D–F.** *C. cerasiformis* NIES-424. Horizontal panels show the same cells, composed of Nomarski differential interference images (**A, D**), epifluorescence images with DAPI staining (**B, E**) and epifluorescence images with the volv-835 probe specific for the endosymbiont of *C. cerasiformis* NIES-425 (**C**, **F**; for details, see Materials and Methods). Arrowheads point to the signals from the endosymbionts. The green signals (**C**) represent endosymbiont-specific probes and the yellow background (**C**, **F**) is autofluorescence. All are shown at the same magnification. The ‘n’ indicates host cell nuclei.

To identify bacteria corresponding to the obtained rickettsiacean sequences, we designed a specific oligonucleotide probe, Volv-853, with helper probes help-volv1 and help-volv2, targeting 16 S *r*RNA (see Materials and Methods). In *C. cerasiformis* NIES-425, endosymbiont-specific signals (Volv-835) were detected exclusively from the rod-shaped bodies within the cytoplasm (Figure 3A–C). These bodies corresponded almost exactly to the rod-shaped light blue fluorescence when visualized by DAPI-staining (Figure 3B). The EUB338MIX (targeting 16 S *r*RNA of eubacteria and chloroplasts, see Materials and Methods) signals showed net-like hybridization patterns within the chloroplasts of *C. cerasiformis* NIES-425 cells (Figure S6A–C). In contrast, within the cells of *C. cerasiformis* NIES-424, no fluorescent signal from probe Volv-835 was detected (Figure 3D–F), while EUB338MIX showed the same net-like pattern as in *C. cerasiformis* NIES-425 (Figure S6D–F). In *P. japonica* NIES-577, the endosymbiont-specific signal of Volv-835 showed rod-shaped bodies corresponding to the cytoplasmic DAPI signals, as seen in *C. cerasiformis* NIES-425 (Figure S7).

### Detection of rickettsiacean 16 S *r*RNA in various strains of four *Carteria* species and *Pleodorina japonica*

Polymerase chain reaction (PCR) using Rickettsiaceae-specific 16 S *r*RNA primers (Table S2) was performed to detect rickettsiacean endosymbionts in ten strains of four *Carteria* species and *P. japonica* NIES-577. A single PCR band representing the presence of rickettsiacean bacteria was detected in *C. cerasiformis* NIES-425 and *P. japonica* NIES-577, while no amplification occurred in the remaining nine *Carteria* strains (Figure S8).

## Discussion

### Characterization of endosymbionts

Our phylogenetic analysis demonstrated that both bacterial endosymbionts from *Carteria cerasiformis* NIES-425 and *Pleodorina japonica* NIES-577 belong to the family Rickettsiaceae (Figure 2). These rickettsiacean endosymbionts are rod-shaped (Figure S2), surrounded by an electron-lucent zone, and either wholly or partly lack a phagosomal membrane in the host cytoplasm [20], [22], as in other species belonging to the Rickettsiaceae [1]. These features are different from those of other families in the order Rickettsiales, such as *Anaplasma* (Anaplasmataceae) or *Holospora* (Holosporaceae) [1], in which bacteria are obviously surrounded by a host cell membrane-derived vacuole (*Anaplasma*) or multiply within the host nucleus (*Holospora*).

### Transmission of endosymbionts to their host cells

Both *C. cerasiformis* strains containing and lacking endosymbionts (NIES-425 and NIES-424, respectively) showed more than 10-fold growth, and typical sigmoid growth curves, over a 192 h period post-inoculation (Figure S5). Thus, the rickettsiacean endosymbionts of volvocalean cells should not be harmful to their hosts. However, the endosymbiont-containing strain showed slightly slower growth than the endosymbiont-lacking strain (Figure S5). This result could be explained by some inhibition of growth of the host *Carteria* cells due to the endosymbionts. Another possible explanation might be physiological and genetic differences between the host cell strains because they originate from different habitats [22], [24], even though they have identical *rbcL* gene sequences [23] (Figure S1). *C. cerasiformis* NIES-424 originates from a large lake (Lake Kasumigaura, Ibaraki, Japan), whereas *C. cerasiformis* NIES-425 was isolated from a water sample collected in a pond (Tsukuba, Ibaraki, Japan) [24].

In *C. cerasiformis* NIES-425, cells of all ages contained rod-shaped bacterial endosymbionts (Figure S2). This permanent coexistence and the positive correlation between host cell size and number of bacterial endosymbionts in *C. cerasiformis* NIES-425 (Figure 1) suggest that the endosymbionts are transmitted to each daughter cell during asexual reproduction of *C. cerasiformis* NIES-425, and that growth of these endosymbionts is likely restricted by the age or volume of the host *Carteria* cells. Similar growth of the endosymbionts was reported in *P. japonica*, within which the number of endosymbionts increased as the host cells matured [20].

### Origin of endosymbionts

Of the four related *Carteria* species, endosymbionts were detected in only *C. cerasiformis* NIES-425 cells by TEM (Figure S1) [22] and DAPI-staining (Figures S2, S3 and Table 1). PCR using Rickettsiaceae-specific primers demonstrated the absence of rickettsiacean DNA within nine other strains of *Carteria* (Figure S8), supporting these observations. In addition, there were markedly fewer differences in the 16 S *r*RNA sequences of rickettsiacean endosymbionts of *C. cerasiformis* NIES-425 and *P. japonica* NIES-577 than in the chloroplast 16 S *r*RNA and 18 S *r*RNA of the host volvocalean cells (Table 2). This remarkable difference in the degree of divergence is indicative of horizontal transfer of the rickettsial endosymbionts, even though the three *r*RNA genes have different mutation rates.

Recent studies have expanded the known diversity of rickettsiacean endosymbionts. Weinert et al. [14] resolved rickettsiacean endosymbionts in various non-arthropod hosts as a large monophyletic group (hydra group, corresponding to group II in Figure 2). Our study showed that endosymbionts of *C. cerasiformis* and *P. japonica* constitute a small clade with endosymbionts of the ciliate *D. appendiculata* and three environmental sequences within group II, and are separated from the typical arthropod-associated rickettsiacean species (Figure 2). *D. appendiculata* was collected from Baltic Sea water and fed marine algae [11], whereas all *Carteria* and *Pleodorina* species grow in freshwater habitats [18], [20], [22]. It is unlikely that rickettsiacean bacteria were transferred to *D. appendiculata* from *Carteria* or *Pleodorina* by direct contact. However, one of the closely related environmental sequences within group II originated from a freshwater lake in China (Figure 2), where volvocalean algae harboring rickettsiacean endosymbionts may grow. Thus, it is possible that non-arthropod species harboring such rickettsiacean endosymbionts are widely distributed in natural freshwater environments, and their endosymbionts may be transmitted to various non-arthropod organisms including volvocalean algae and ciliates.

However, horizontal transmission of rickettsiacean bacteria has been observed only from arthropods to vertebrates [25], except for one probable case from an arthropod (leafhopper) to a land plant (papaya) [17]. In general, rickettsiacean infection involves two steps: adhesion to the host cell surface and invasion by phagocytosis [3]. Volvocalean algae have not been reported to be phagotrophic, and little is known about endocytosis in green algae [26]. Furthermore, volvocalean algae cells possess a cell wall or extracellular matrix [18] that might inhibit bacterial invasion. However, during sexual reproduction, *Carteria* and *Pleodorina* produce naked gametes released from the parent [20], [22]. Therefore, infection of endosymbionts during sexual reproduction may occur via unknown mechanisms that will be the focus of future studies.

### Nomenclature of endosymbionts

Rickettsiacean endosymbionts within both volvocalean species were categorized as bacterial species [27] because they exhibited an only 0.6% difference in 16 S *r*RNA gene sequences (Table 2). Furthermore, they belonged to group II (the hydra group) and had similarity values of 92.5–93.6% with the sequences of bacteria in group I, which is sister to group II (Figure 2). No other endosymbionts/sequences within the hydra group have valid or provisional taxonomic names [14]. However, phylogenetic positions of the endosymbionts harboring in the green algal and ciliate cells within the hydra group were not well resolved in the present 16 S *r*RNA gene phylogeny (Figure 2). Thus, we postpone the proposal of a new candidate species for these endosymbionts until additional evidence has been gathered.

### Conclusions

These data suggest that the rickettsiacean endosymbionts are not harmful to their green algal hosts and might have been transmitted recently from non-arthropod organisms. Because rickettsias are closely related to mitochondria [5], incipient stages of mitochondrial endosymbiosis will be deduced by studying both *C. cerasiformis* strains with and without endosymbionts. Furthermore, these strains might be available for future studies to resolve the medical problems caused by rickettsial infection.

## Materials and Methods

### Cultures and growth measurements

All volvocalean strains used in this study were supplied from the Microbial Culture Collection at the National Institute for Environmental Studies, Japan (NIES) [24] (Table 1). The cultures were grown in screw-cap tubes (18×150 mm; Fujimoto Rika, Tokyo, Japan) containing 10 ml AF-6 medium [28] modified as described by Kasai et al. [24]. The cultures were maintained at 20°C, under irradiance with ca. 100–150 µmol photons m-2s-1, with a 14:10 h light-dark (LD) photoperiod provided by cool-white fluorescent lamps. To detect contaminating bacteria, each strain was inoculated into B-V medium [29], and cultured at 37°C in darkness for 1 week. No bacterial growth was detected in DAPI-stained cells of any strain under epifluorescence microscopy.

For growth measurement, ∼105 cells of an actively growing, 6-day-old culture in AF-6 medium (0.8–1.1 ml) were inoculated into new AF-6 medium (10 ml). The cultures were grown at 20°C, under irradiance with 126 µmol photons m-2s-1, with a 14:10 h LD photoperiod provided by cool-white fluorescent lamps. Growth of each culture tube was measured after 48, 96, 144, and 192 h, by counting cell numbers, as follows: 1 ml homogenously mixed culture was mixed with 40 µl 25% glutaraldehyde for 15 min at room temperature. The fixed cells were concentrated by centrifugation (20,400 *g*). The volumes were determined and the cells were counted using a Fuchs-Rosenthal counting chamber (Fuji Rika Kogyo, Osaka, Japan). This experiment was performed on three replicate cultures.

### DAPI staining

For DAPI staining, an actively growing, 6- to 8-day-old culture was fixed for 30 min with a final concentration of 1% glutaraldehyde at room temperature. Fixed materials were added to an equal volume of DAPI solution (1 mg/ml) in NS buffer [30] and squashed by pushing the cover glass onto the cells. These materials were observed under an epifluorescence microscope (BX-60, Olympus, Tokyo, Japan) equipped with Nomarski interference.

To examine the relationship between the size of host *Carteria* cells and the number of bacterial endosymbionts, we used 6- to 7-day-old cultured cells fixed for 1, 8, and 14 h from the beginning of the light period. The number of endosymbionts was determined by DAPI staining, as described above. Host cell size was expressed as the diameter of squashed, circular cells, and the sizes in a single preparation were compared and plotted on a single graph.

### PCR amplification and sequencing of genes

Cultured cells were boiled for 5 min before disruption. Subsequently, the modified protocol [31] of Fawley and Fawley [32] was used to extract total DNA from 11 strains (Table 1).

For sequencing of bacterial 16S *r*RNA genes, PCR was performed using the total DNA of *C. cerasiformis* NIES-425 and *Pleodorina japonica* NIES-577 and universal primers (9F and 1492R; Table S2), with TaKaRa *Taq* polymerase (Takara Bio, Shiga, Japan), as described previously [33]. *C. cerasiformis* NIES-425 showed two bands of different sizes (Figure S9) corresponding to the expected size of 16 S *r*RNA (ca. 1.4 kbp), possibly from the endosymbionts, and chloroplast *r*RNA interrupted by a putative group I intron [34], [35]. However, *P. japonica* NIES-577 showed a single band of ca. 1.4 kbp (Figure S9) from which only the chloroplast 16 S *r*RNA gene sequence was detected by direct sequencing (not shown). PCR products from the *C. cerasiformis* NIES-425 bacterial endosymbionts were then cloned into a plasmid vector (pCR®4-TOPO®) using a TOPO TA Cloning Kit for Sequencing (Invitrogen, Carlsbad, CA, USA). At least five clones from each sample were randomly chosen and sequenced as described previously [33], [36]. The 16 S *r*RNA sequence of the endosymbiont of *C. cerasiformis* NIES-425 was extended in both directions, based on the direct sequencing methodology with specific primers, as described previously [36]. Specific primers for the 16 S *r*RNA of Rickettsiaceae (Table S2) were designed using the partial sequences obtained from the cloned PCR products and published 16 S *r*RNA of related, rickettsiacean bacteria.

The *P. japonica* NIES-577 endosymbiont 16 S *r*RNA sequence was determined, based on the direct sequencing methodology with specific primers for the 16 S *r*RNA of Rickettsiaceae (Table S2), as described previously [36]. Chloroplast 16 S *r*RNA and nuclear 181S *r*RNA sequences of *C. cerasiformis* NIES-425 and *P. japonica* NIES-577 were determined using the PCR primers listed in Table S2 and the direct sequencing method described above.

To detect the rickettsiacean endosymbionts, genomic PCR was carried out using two of the specific primers for the 16 S *r*RNA of Rickettsiaceae (N577enFE and N577enRG, Table S2) and two for the 18 S *r*RNA (FA and RF, Table S2), as described previously [36].

### Phylogenetic analysis

The 16 S *r*RNA genes of two bacterial endosymbionts of *C. cerasiformis* NIES-425 and *P. japonica* NIES-577 and 45 related bacterial and environmental sequences (Table S1) were aligned in ARB software [37]. The alignment was corrected manually referring to the secondary structure. A maximum likelihood phylogenetic analysis with the TrN [38]+gamma+I model (selected by MODELTEST 3.06 [39]) was carried out using two programs: PhyML [40] and PAUP* 4.0b10 [41]. The maximum parsimony (MP) method was performed using the tree-bisection-reconnection (TBR) branch-swapping algorithm in PAUP* 4.0b10 [41]. The bootstrap values were calculated for 1000 replications. For the analysis of chloroplast 16 S *r*RNA and nuclear 18 S *r*RNA sequences, CLUSTAL X [42] was used for alignment with the default options.

### *In situ* hybridization

Volv-835 (Table S2), an oligonucleotide probe targeting the 16 S *r*RNA of bacterial endosymbionts of *C. cerasiformis* NIES-425, was designed using the probe-designing function of ARB. The sequence specificity of the probe was checked by probeCheck [43], and the optimal temperature and formamide concentration for specific hybridization was estimated using DINAMelt [44]. Volv-835 did not match the host's chloroplast 16 S *r*RNA *in silico*. In the SILVA database [45], probe Volv-835 matched only five 16 S *r*RNA sequences, including that of an endosymbiont of *D. appendiculata* with 99% identity to the endosymbionts of the volvocalean species; the other sequences contained at least two mismatches. EUB338MIX, which is a mixture of the probes EUB338 [46], EUB338-II [47], and -III [47], was used (Table S2) to detect a wide-range of eubacterial taxa. EUB338 matches the 16 S *r*RNA sequences of the endosymbionts of *C. cerasiformis* NIES-425 and *P. japonica* NIES-577, and their chloroplast 16 S *r*RNA sequences *in silico*. These probes were labeled with 6-carboxyfluorescein at their 5′ end. Two non-labeled oligonucleotides were also designed and used as helper probes [48] to Volv-835: help-volv1 and help-volv2 (Table S2).

FISH was performed according to the method of Noda *et al.* [49] with some modifications. Actively growing, 5- to 7-day-old cultures in AF-6 medium were fixed with 4% paraformaldehyde in phosphate-buffered saline (PBS) overnight at 4°C and washed in PBS twice. Fixed cells were incubated with enzyme solution (1% cellulase Onozuka RS, 1% macerozyme R-10 [Yakult Pharmaceutical industry, Tokyo, Japan] in PBS) for 5 min, washed in PBS, and suspended in 1% Tween 20 (Sigma-Aldrich, Missouri, U.S.) solution in PBS. After washing in PBS twice, the treated cells were spotted on an aminosilane-coated glass slide (Matsunami Glass, Osaka, Japan), air-dried, and treated with 0.25N HCl for 20 min at room temperature. Samples were then washed with distilled water and sequentially dehydrated in 50, 80, 90, and 100% ethanol. Oligonucleotide probes with 20% formamide in the hybridization buffer (0.1 M Tris-HCl, 0.9 M NaCl) were then applied, sealed in an incubation chamber (CoverWell; Grace Bio-Labs, Oregon, U.S.), and incubated for 90 min at 51°C. After washing in wash buffer (0.1 M Tris-HCl, 0.2 M NaCl) and high stringency wash buffer (20 mM Tris-HCl, 40 mM NaCl), slides were mounted with enclosing liquid (90% glycerol, 1% triethylenediamine in PBS) containing DAPI, and observed under an epifluorescence microscope, the Olympus BX-60.

## Supporting Information

Figure S1 **Phylogenetic distribution of bacterial endosymbionts in various strains of** ***Carteria cerasiformis*** **and related species.** The phylogeny was redrawn based on *rbcL* genes [23], by maximum parsimonious analysis using the TBR branch-swapping algorithm with bootstrap analysis on 1000 replicates using the program PAUP* 4.0b10 [41]. Presence (○) or absence (▴) of bacterial endosymbionts is based on the transmission electron microscopy by Nozaki et al. [22]. Asterisks indicate strains examined in this study.
(TIF)

Figure S2 **DAPI-stained** ***Carteria cerasiformis*** **NIES-425 cells.** **A–C**. Immature cells. **D–F**. Mature cells. Horizontal panels show the same cells, composed of Nomarski differential interference images (**A, D**), epifluorescence images at the periphery of the cytoplasm (**B, E**), and epifluorescence images at the optical section (**C, F**). All are shown at the same magnification. The arrowhead, arrow, ‘ch’ and ‘n’ indicate the bacterial endosymbiont, the chloroplast nucleoid, the chloroplast and the host nuclei, respectively.
(TIF)

Figure S3 **Cells of two** ***Carteria*** **strains stained with DAPI.** **A–C**. *C. inversa* NIES-422. **D–F**. *C. cerasiformis* NIES-424. Horizontal panels show the same cells, composed of Nomarski differential interference images (**A, D**), epifluorescence images at the periphery of the cytoplasm (**B, E**), and epifluorescence images of an optical section (**C, F**). All are shown at the same magnification. The arrow, ‘ch’ and ‘n’ indicate the chloroplast nucleoid, the chloroplast and the host nuclei, respectively.
(TIF)

Figure S4 **Comparison between host cell size and the number of endosymbionts in** ***Carteria cerasiformis*** **NIES-425.** Details are described in the legend to Figure 1. **A.** Cells fixed after 8 hours from beginning of light period, N = 105, Pearson correlation coefficient (*r*) = 0.76. **B.** Cells fixed after 1 hour from beginning of light period, N = 89, *r* = 0.80.
(TIF)

Figure S5 **Comparison of growths between** ***Carteria cerasiformis*** **NIES-425 (with bacterial endosymbionts) and NIES-424 (without bacterial endosymbionts).** Vertical axis represents common logarithm (log_10_) of cell numbers per one culture tube (see Materials and Methods in the text).
(TIF)

Figure S6 **FISH images with EUB338MIX in vegetable cells of two** ***Carteria cerasiformis*** **strains.** **A–C.** *C. cerasiformis* NIES-425. **D–F.** *C. cerasiformis* NIES-424. Horizontal panels show the same cells, composed of Nomarski differential interference images (**A, D**), epifluorescence images with DAPI staining (**B, E**; n, the host cell nuclei) and epifluorescence images with 16 S *r*RNA probes EUB338 MIX (see Materials and Methods) (**C, F**). All are shown at the same magnification.
(TIF)

Figure S7 **FISH identification of the rickettsiacean endosymbionts in** ***Pleodorina japonica*** **NIES-577 cells.** Horizontal panels show the same cells, composed of Nomarski differential interference image (**A**), epifluorescence image with DAPI staining (**B**) and epifluorescence image with the probe volv-835, specific for the endosymbionts of *P. japonica* NIES-577 (**C**; for details, see Materials and Methods in the text). Arrowheads point to the signals from the endosymbionts. In **C**, green signals represent the endosymbiont-specific probes and yellow background represents autofluorescence. All are shown at the same magnification.
(TIF)

Figure S8 **Detection of rickettsiacean 16 S** ***r*** **RNA in various strains of four** ***Carteria*** **species and** ***Pleodorina japonica*** **.** PCR amplification by Rickettsiaceae-specific 16 S *r*RNA primers N577enFE and N577enRG (Table S2) shows the presence or absence of rickettsiacean endosymbionts. The eukaryotic 18 S rRNA gene was amplified by primers FA and RF (Table S2) as a control.
(TIF)

Figure S9 **PCR with bacterial universal 16 S** ***r*** **RNA primers in two volvocalean species with bacterial endosymbionts.** Arrowhead indicates expected size (ca. 1.4 kbp) of the amplified DNA fragment of only coding region of 16 S *r*RNA by two primers (9F and 1492R; Table S2). Longer fragment (ca. 2.3 kbp, indicated by arrow) in *Carteria cerasiformis* NIES-425 represents the presence of interrupted group I intron in the chloroplast 16 S *r*RNA. *Pleodorina japonica* NIES-577 shows only a single-sized fragment (ca. 1.4 kbp).
(TIF)

Table S1 **List of bacterial 16S** ***r*** **RNA gene sequences used in this study.**
(DOC)

Table S2 **Primers and probes used in this study** [**31]**, [46], [47], [51], [52****] **.**
(DOC)
