# Supplementary figures and images for: Molecular Identification of Rickettsial Endosymbionts in the Non-Phagotrophic Volvocalean Green Algae
Source: PLoS One. 2012 Feb 21;7(2):e31749. doi: 10.1371/journal.pone.0031749 (PMC3283676; doi:10.1371/journal.pone.0031749)

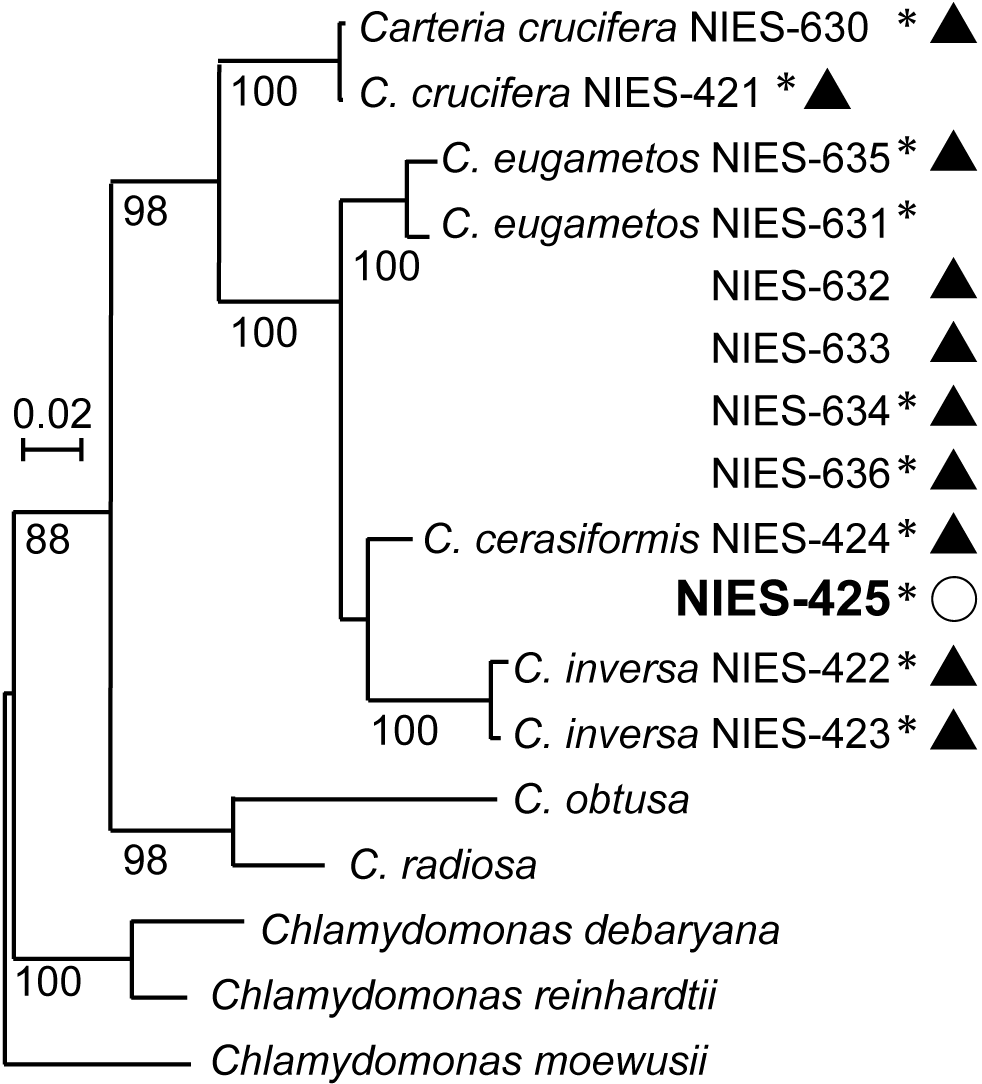

Supplement: Figure S1 — Phylogenetic distribution of bacterial endosymbionts in various strains of Carteria cerasiformis and related species. The phylogeny was redrawn based on rbcL genes [23], by maximum parsimonious analysis using the TBR branch-swapping algorithm with bootstrap analysis on 1000 replicates using the program PAUP* 4.0b10 [41]. Presence (○) or absence (▴) of bacterial endosymbionts is based on the transmission electron microscopy by Nozaki et al. [22]. Asterisks indicate strains examined in this study. (TIF) [file pone.0031749.s001.tif]

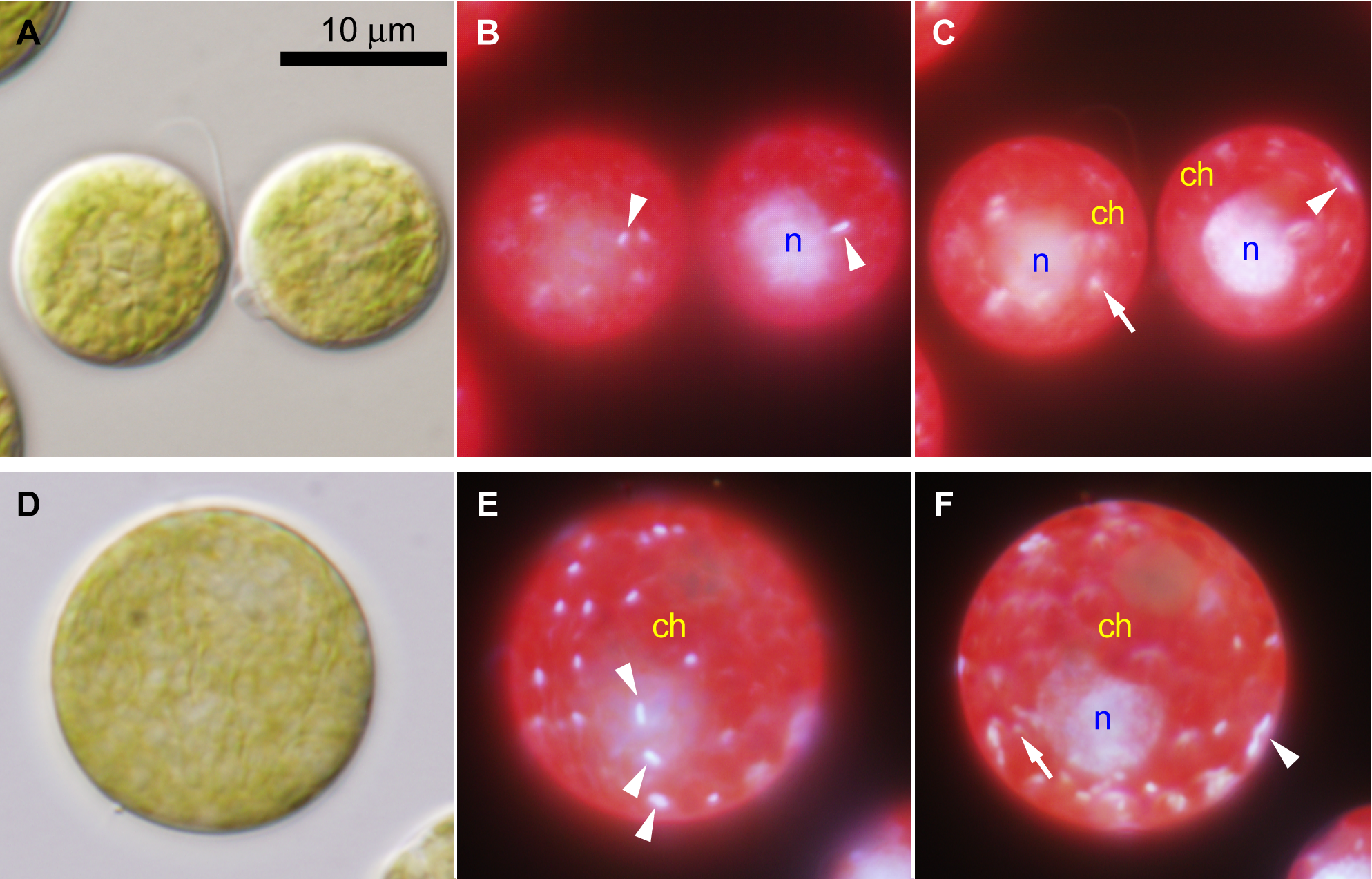

Supplement: Figure S2 — DAPI-stained Carteria cerasiformis NIES-425 cells. A–C. Immature cells. D–F. Mature cells. Horizontal panels show the same cells, composed of Nomarski differential interference images (A, D), epifluorescence images at the periphery of the cytoplasm (B, E), and epifluorescence images at the optical section (C, F). All are shown at the same magnification. The arrowhead, arrow, ‘ch’ and ‘n’ indicate the bacterial endosymbiont, the chloroplast nucleoid, the chloroplast and the host nuclei, respectively. (TIF) [file pone.0031749.s002.tif]

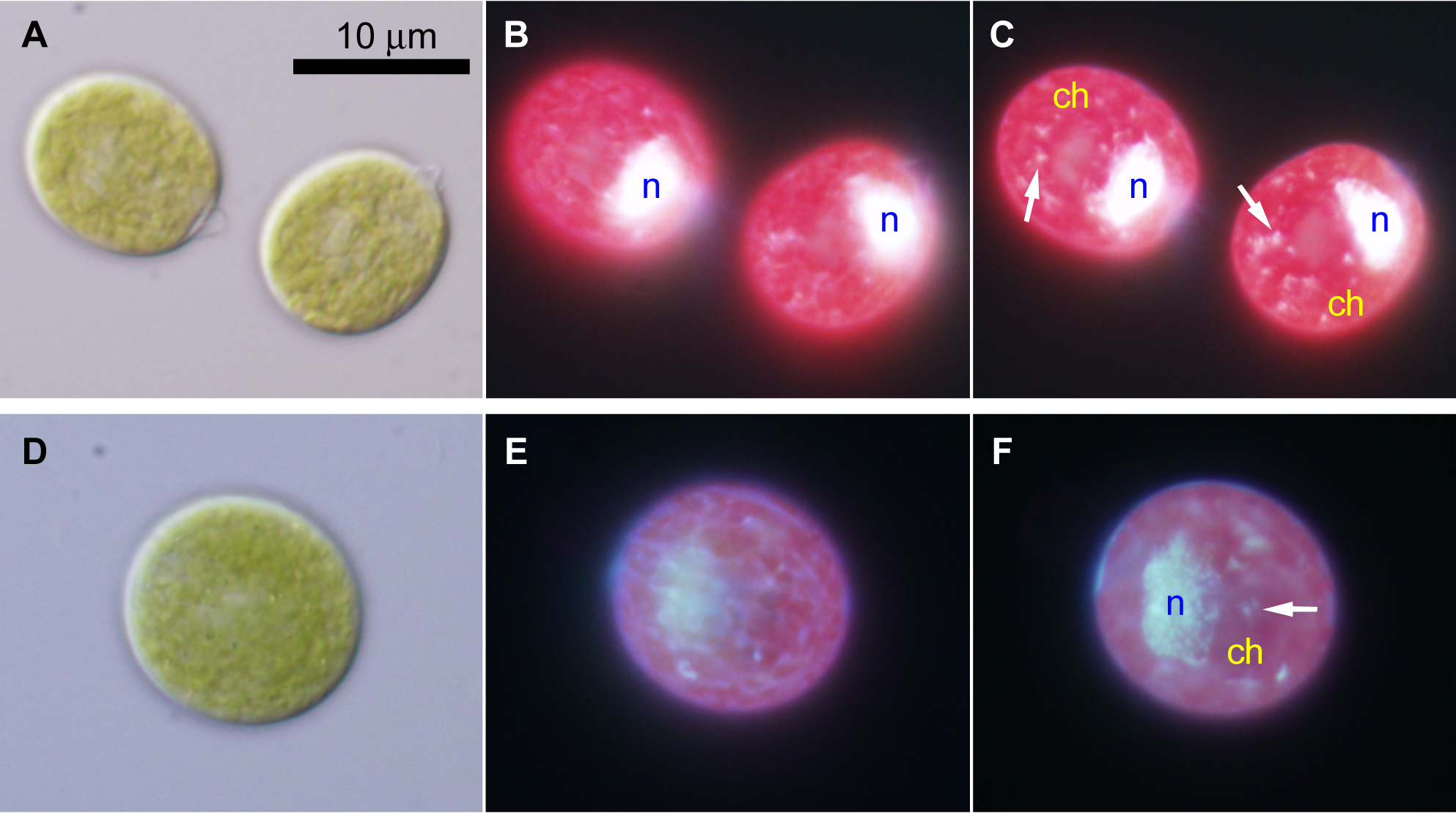

Supplement: Figure S3 — Cells of two Carteria strains stained with DAPI. A–C. C. inversa NIES-422. D–F. C. cerasiformis NIES-424. Horizontal panels show the same cells, composed of Nomarski differential interference images (A, D), epifluorescence images at the periphery of the cytoplasm (B, E), and epifluorescence images of an optical section (C, F). All are shown at the same magnification. The arrow, ‘ch’ and ‘n’ indicate the chloroplast nucleoid, the chloroplast and the host nuclei, respectively. (TIF) [file pone.0031749.s003.tif]

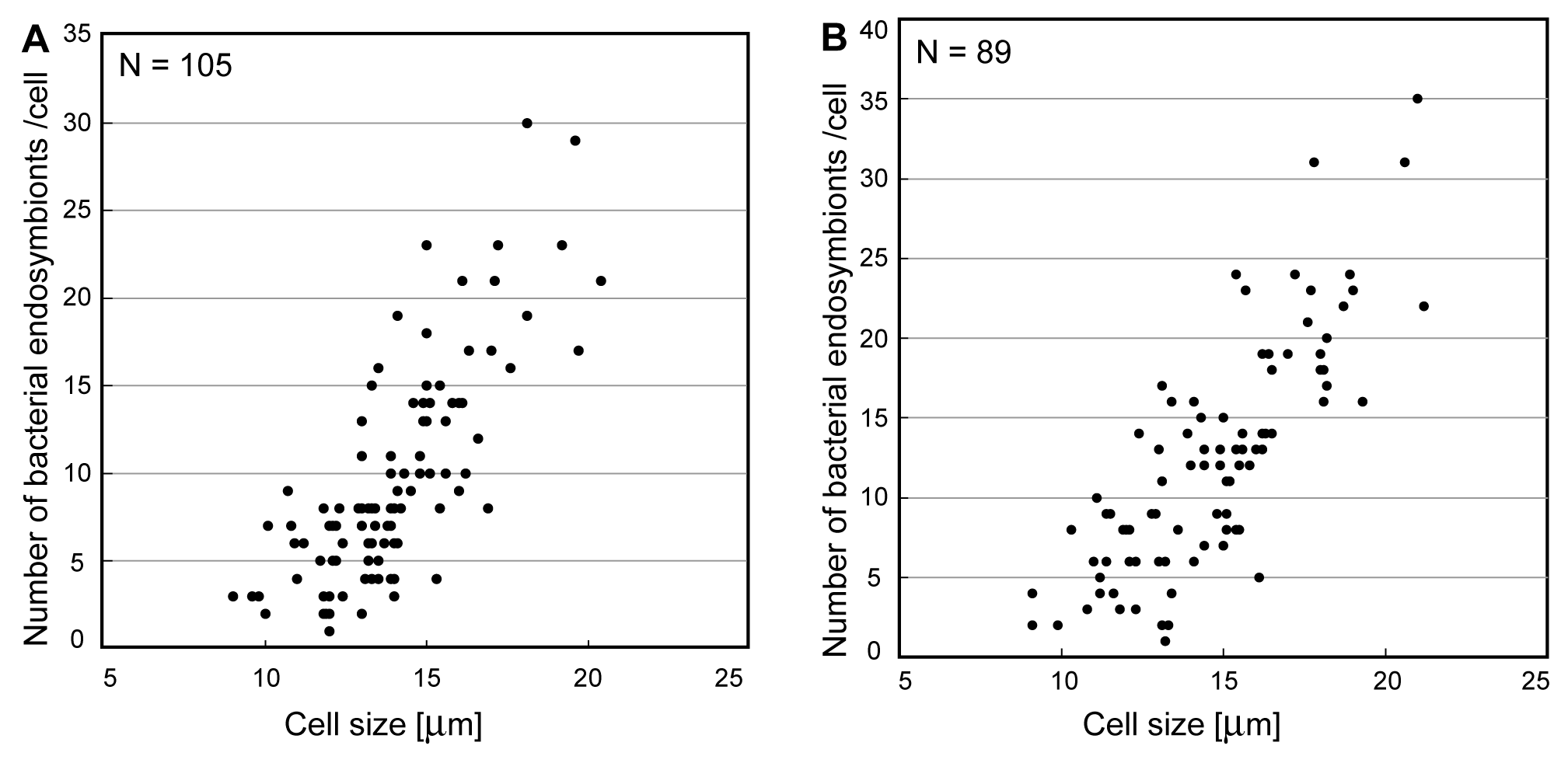

Supplement: Figure S4 — Comparison between host cell size and the number of endosymbionts in Carteria cerasiformis NIES-425. Details are described in the legend to Figure 1. A. Cells fixed after 8 hours from beginning of light period, N = 105, Pearson correlation coefficient (r) = 0.76. B. Cells fixed after 1 hour from beginning of light period, N = 89, r = 0.80. (TIF) [file pone.0031749.s004.tif]

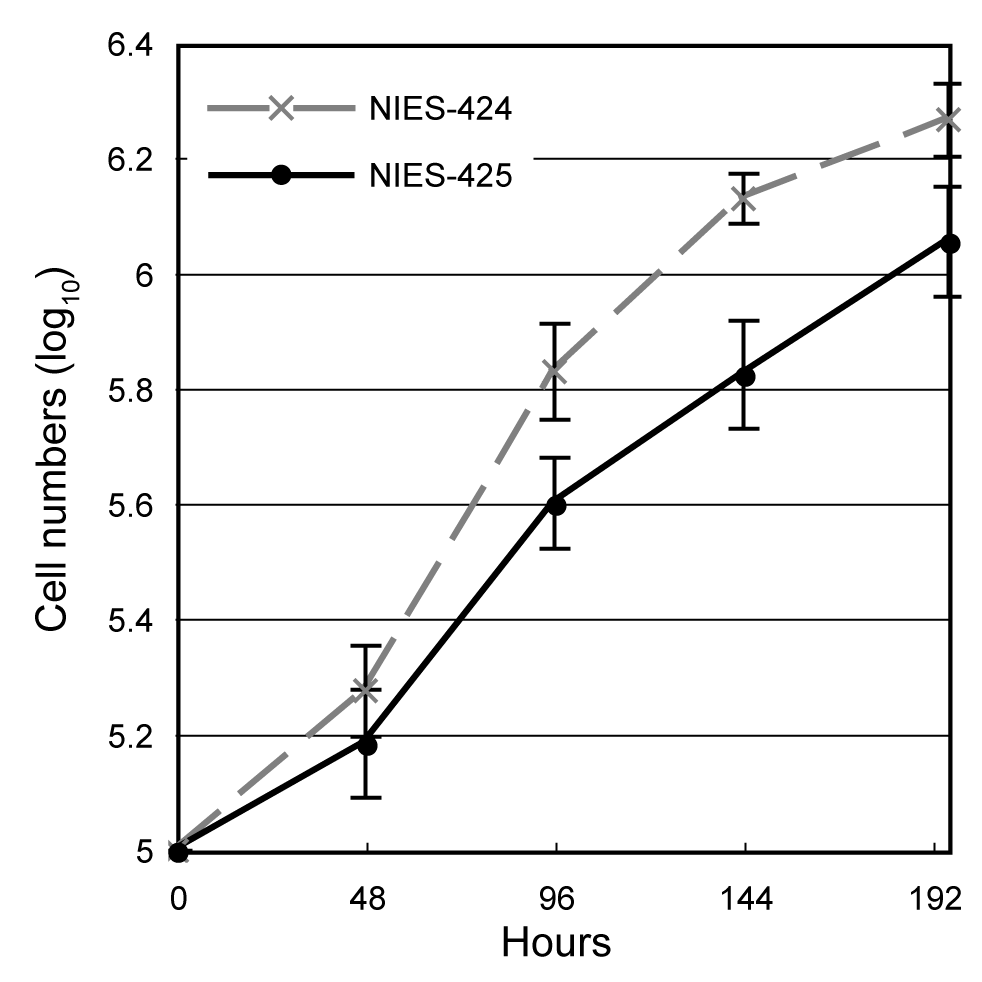

Supplement: Figure S5 — Comparison of growths between Carteria cerasiformis NIES-425 (with bacterial endosymbionts) and NIES-424 (without bacterial endosymbionts). Vertical axis represents common logarithm (log10) of cell numbers per one culture tube (see Materials and Methods in the text). (TIF) [file pone.0031749.s005.tif]

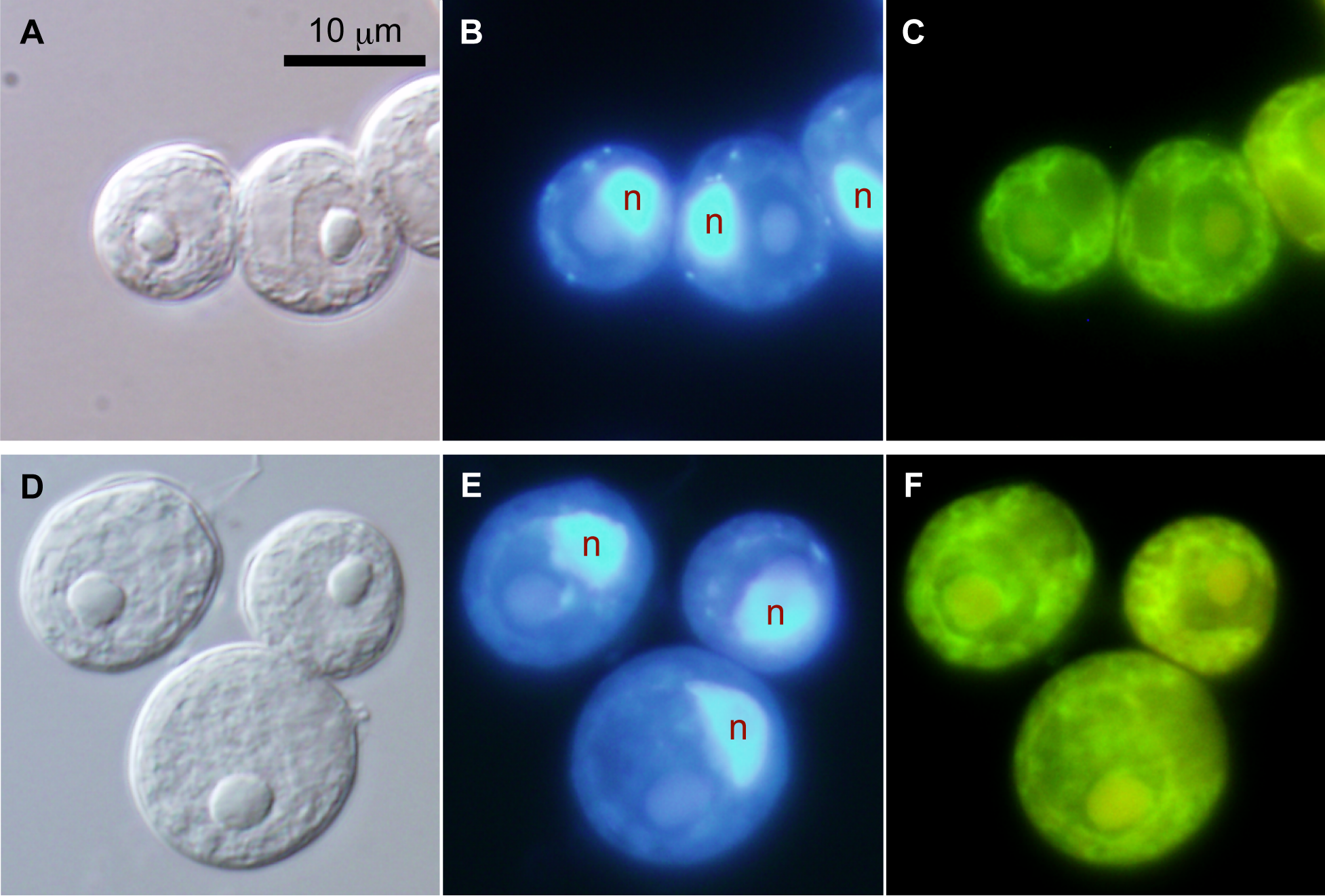

Supplement: Figure S6 — FISH images with EUB338MIX in vegetable cells of two Carteria cerasiformis strains. A–C. C. cerasiformis NIES-425. D–F. C. cerasiformis NIES-424. Horizontal panels show the same cells, composed of Nomarski differential interference images (A, D), epifluorescence images with DAPI staining (B, E; n, the host cell nuclei) and epifluorescence images with 16 S rRNA probes EUB338 MIX (see Materials and Methods) (C, F). All are shown at the same magnification. (TIF) [file pone.0031749.s006.tif]

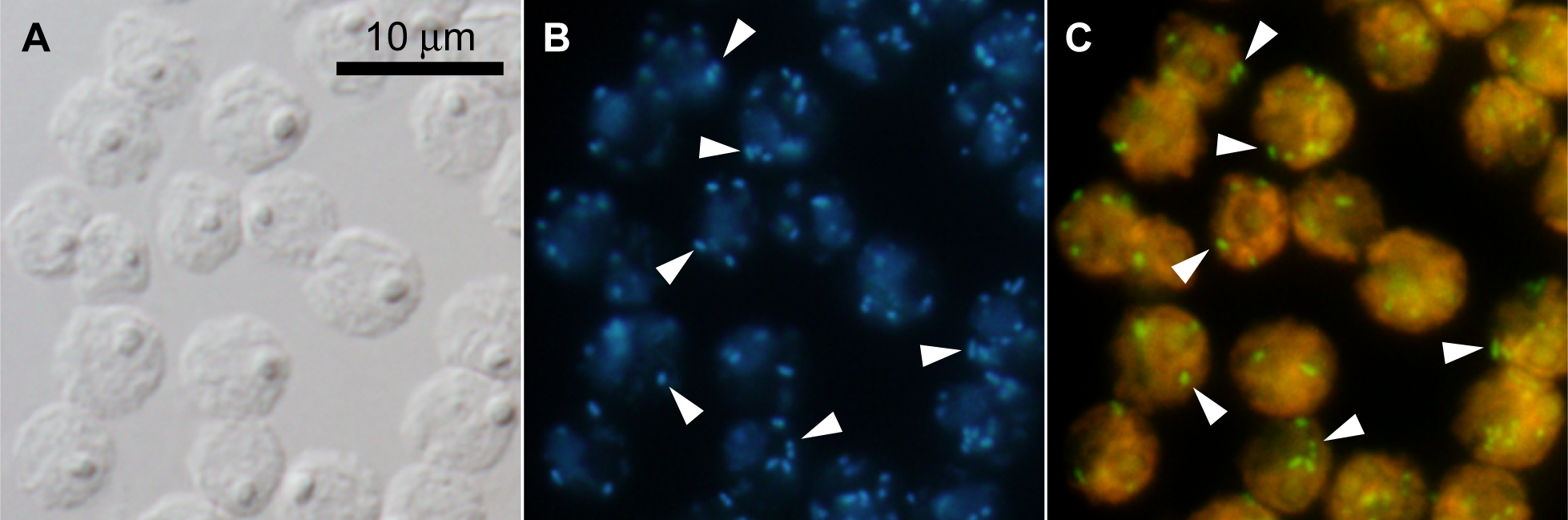

Supplement: Figure S7 — FISH identification of the rickettsiacean endosymbionts in Pleodorina japonica NIES-577 cells. Horizontal panels show the same cells, composed of Nomarski differential interference image (A), epifluorescence image with DAPI staining (B) and epifluorescence image with the probe volv-835, specific for the endosymbionts of P. japonica NIES-577 (C; for details, see Materials and Methods in the text). Arrowheads point to the signals from the endosymbionts. In C, green signals represent the endosymbiont-specific probes and yellow background represents autofluorescence. All are shown at the same magnification. (TIF) [file pone.0031749.s007.tif]

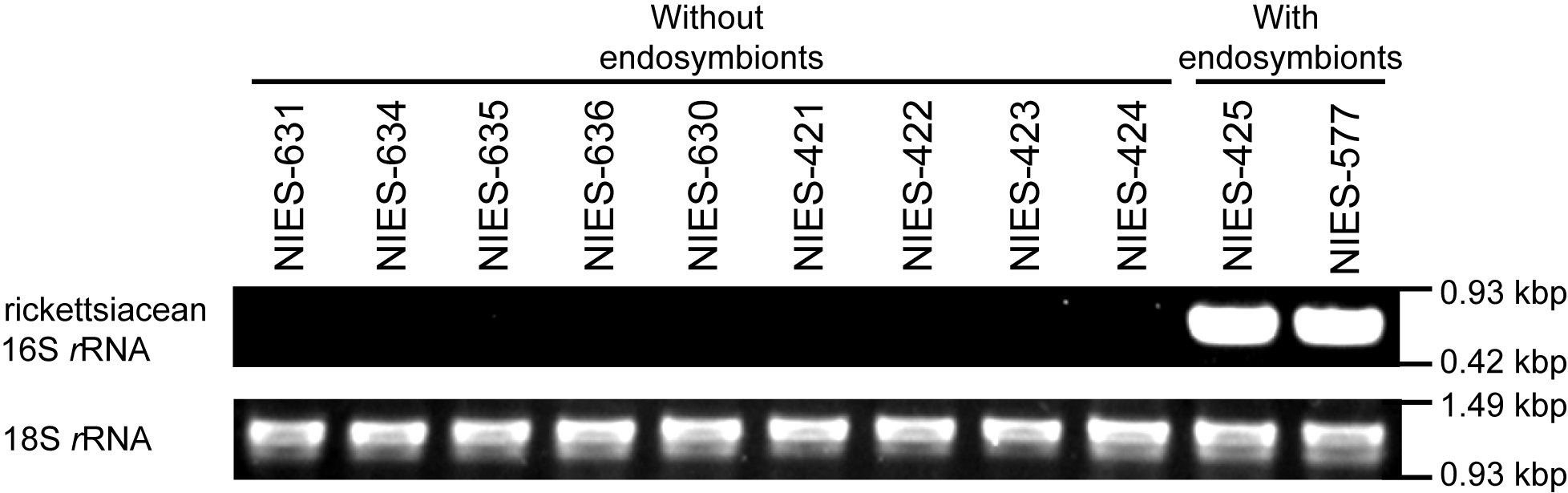

Supplement: Figure S8 — Detection of rickettsiacean 16 S r RNA in various strains of four Carteria species and Pleodorina japonica . PCR amplification by Rickettsiaceae-specific 16 S rRNA primers N577enFE and N577enRG (Table S2) shows the presence or absence of rickettsiacean endosymbionts. The eukaryotic 18 S rRNA gene was amplified by primers FA and RF (Table S2) as a control. (TIF) [file pone.0031749.s008.tif]

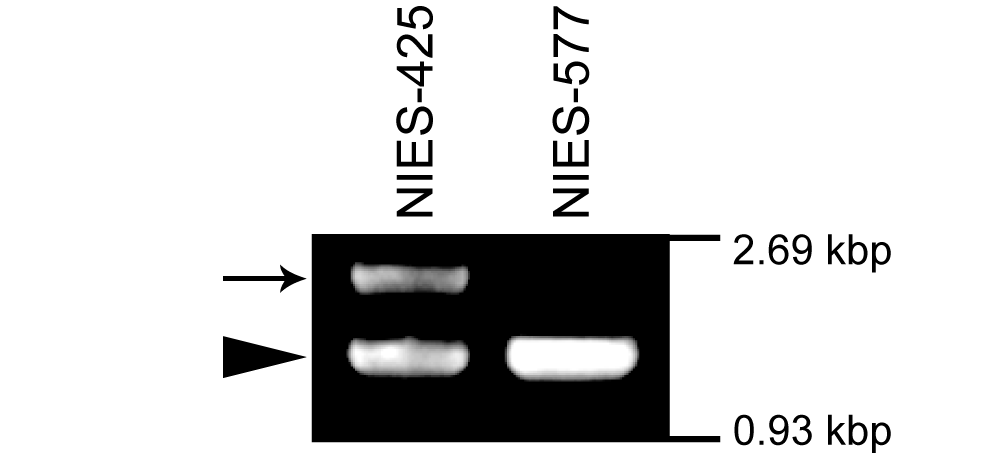

Supplement: Figure S9 — PCR with bacterial universal 16 S r RNA primers in two volvocalean species with bacterial endosymbionts. Arrowhead indicates expected size (ca. 1.4 kbp) of the amplified DNA fragment of only coding region of 16 S rRNA by two primers (9F and 1492R; Table S2). Longer fragment (ca. 2.3 kbp, indicated by arrow) in Carteria cerasiformis NIES-425 represents the presence of interrupted group I intron in the chloroplast 16 S rRNA. Pleodorina japonica NIES-577 shows only a single-sized fragment (ca. 1.4 kbp). (TIF) [file pone.0031749.s009.tif]
