# Supplementary material for: Molecular Identification of Rickettsial Endosymbionts in the Non-Phagotrophic Volvocalean Green Algae
Source: PLoS One. 2012 Feb 21;7(2):e31749. doi: 10.1371/journal.pone.0031749 (PMC3283676; doi:10.1371/journal.pone.0031749)
Supplement: Table S1 — List of bacterial 16S r RNA gene sequences used in this study. (DOC) [file pone.0031749.s010.doc]

**Table S1. List of bacterial 16S *r*RNA gene sequences used in this study.**

| **Taxon** | **DDBJ/EMBL/Genbank Accession no.** |
| --- | --- |
| *Anaplasma marginale* | AY048816 |
| *Anaplasma platys* | M82801 |
| *Candidatus* Anadelfobacter veles | FN552695 |
| *Candidatus* Cryptoprodotis polytropus (endosymbiont of *Pseudomicrothorax dubius*) | FM201293 |
| *Candidatus* Cyrtobacter comes | FN552698 |
| *Candidatus* Midichloria mitochondrii | AJ566640.2 |
| *Candidatus* Odyssella thessalonicensis | AF069496 |
| *Ehrlichia canis* | M73221 |
| *Ehrlichia ruminantium* | AF069758 |
| Endosymbiont of *Acanthamoeba* polyphaga HN-3 | AF132138 |
| Endosymbiont of *Acanthamoeba* sp. clone UWC8 | AF069963 |
| Endosymbiont of *Acanthamoeba* sp. TUMK-23 | AY102614 |
| Endosymbiont of *Diophrys appendiculata* | AJ630204 |
| Endosymbiont of *Empoasca papayae* | U76910 |
| Endosymbiont of Hemicrepsis marginata | AB066352 |
| Endosymbiont of *Hydra oligatics* | EF667896 |
| Endosymbiont of *Ichthyophthirius multifiliis* | GQ870455 |
| Endosymbiont of *Onchocerca ochengi* | AJ010276 |
| Endosymbiont of *Petalomonas sphagnophila* R1-FM1 | GU477308 |
| Endosymbiont of *Petalomonas sphagnophila* R1-Liz1 | GU477312 |
| Endosymbiont of *Petalomonas sphagnophila* R2-FM3 | GU477314 |
| Endosymbiont of *Petalomonas sphagnophila* R2-Liz3 | GU477316 |
| Endosymbiont of *Torix tagoi* | AB066351 |
| Endosymbiont of *Torix tsukubana* | AB113214 |
| *Holospora obtusa* | X58198 |
| *Neorickettsia risticii* | M21290 |
| *Neorickettsia sennetsu* | NC_007798 |
| *Orientia tsutsugmushi* | D38623 |
| *Rickettsia akari* | CP000847 |
| *Rickettsia australis* | L36101 |
| *Rickettsia bellii* | CP000087 |
| *Rickettsia canadensis* | L36104 |
| *Rickettsia conorii* | L36105 |
| *Rickettsia felis* | CP000053 |
| *Rickettsia helvetica* | L36212 |
| *Rickettsia japonica* | L36213 |
| *Rickettsia limoniae* | AF322442 |
| *Rickettsia prowazekii* | AJ235272 |
| *Rickettsia rickettsii* | U11021 |
| *Rickettsia typhi* | L36221 |
| Uncultured clone (Antarctic hypersaline lake water clone ELB16-030)^a^ | DQ015802 |
| Uncultured clone (Chinise freshwater lake) ^a^ | FJ612282 |
| Uncultured clone (Kalahari Shield South Africa subsurface water clone EV221H2111601SAH71) ^a^ | DQ223223 |
| Uncultured clone (U.S. acid-impacted lake clone ADK-MOe02-3) ^a^ | EF520417 |
| *Wolbachia pipientis* | U23709 |
| Endosymbiont of *Carteria cerasiformis* NIES-425 | AB688628 ^b^ |
| Endosymbiont of *Pleodorina japonica* NIES-577 | AB688629 ^b^ |

^a^Based on the description of GenBank nucleotide collection (National Center of Biotechnology Information; http://www.ncbi.nlm.nih.gov/).

^b^Sequenced in this study.
