# Supplementary material for: Molecular Identification of Rickettsial Endosymbionts in the Non-Phagotrophic Volvocalean Green Algae
Source: PLoS One. 2012 Feb 21;7(2):e31749. doi: 10.1371/journal.pone.0031749 (PMC3283676; doi:10.1371/journal.pone.0031749)
Supplement: Table S2 — Primers and probes used in this study [31] , [46], [47], [51], [52] . (DOC) [file pone.0031749.s011.doc]

**Table S2. Primers and probes used in this study.**

| **Gene** | **Primer/probe name** | **Primer/probe sequence (5'-3')** | **Reference** |
| --- | --- | --- | --- |
| 18S *r*RNA | FA ^a^ | AACCTGGTTGATCCTGCCAGT | 51 |
|  | RD ^b^ | GCTGGCACCAGACTTGCCCTC | 51 |
|  | FG ^a^ | AGTCTGGTGCCAGCAGCCGCG | 51 |
|  | FE ^a^ | GGGAGTATGGTCGCAAGGCTG | 51 |
|  | RF ^b^ | CCCGTGTTGAGTCAAATTAAG | 51 |
|  | RB ^b^ | TGATCCTTCTGCAGGTTCACCTAC | 51 |
|  | FC ^a^ | GGGAGGTAGTGACAAIAAATA | 31 |
| Chloroplast 16S *r*RNA (specific to *Carteria cerasiformis* NIES-425) | N425cpFA ^a^ | ATTGGGCGTAAAGCGTCTGT | this study |
|  | N425cpRB ^b^ | AACAAACTTCGAGGGTTGCG | this study |
| Bacterial 16S *r*RNA | 9F ^a^ | GAGTTTGATCCTGGCTCAG | 52 |
|  | 1492R ^b^ | GCTTACCTTGTTACGACTT | 52 |
|  | EUB338 ^c^ | ACTCCTACGGGAGGCAGC | 46 |
|  | EUB338-II ^c^ | ACACCTACGGGTGGCTGC | 47 |
|  | EUB338-III ^c^ | ACACCTACGGGTGGCAGC | 47 |
| Bacterial 16S *r*RNA (specific to Rickettsiaceae) | N425enFA ^a^ | AGTGGCAAACGGGTGAGTAACA | this study |
|  | N425enRB ^b^ | TAGCTCACCAGCTTCGGGTAAA | this study |
|  | N577enFE ^a^ | CGCGTAGGCGGATTAGTAAGTTGG | this study |
|  | N577enRG ^b^ | CGTCTTGCTTCCCTCTGTAAGT | this study |
| Bacterial 16S *r*RNA (specific to the endosymbionts) | volv-835 ^c^ | CCGAAAGAAAAACTCCCG | this study |
|  | help-volv1 ^d^ | ATATCTAGCACTCATCGT | this study |
|  | help-volv2 ^d^ | AACGTGTTAACTACGAAA | this study |

^a^Forward primer.

^b^Reverse primer.

^c^Fluorecent probe for FISH experiments.

^d^Helper probe for FISH experiments. For details, see the materials and methods.
